# fNIRS evidence of abnormal frontotemporal cortex activation and functional connectivity in depressed patients after stroke: neuromodulatory mechanisms from mild to moderate depression

**DOI:** 10.3389/fneur.2025.1599733

**Published:** 2025-07-18

**Authors:** Shanshan Zhou, Xiaodie Liu, Mengyuan Chen, Wenyi Chen, Yawen Pan, Yinghao Zhi

**Affiliations:** Department of Rehabilitation Medicine, Wenzhou TCM Hospital of Zhejiang Chinese Medical University, Wenzhou, Zhejiang, China

**Keywords:** post-stroke depression, fNIRS, brain activation, functional connectivity, frontotemporal cortex

## Abstract

**Background:**

Post-stroke depression (PSD) is a prevalent psychiatric complication following a stroke, significantly delaying neurological recovery. The assessment of scales in clinical diagnosis often lacks objectivity, while functional near-infrared spectroscopy (fNIRS) has been recognized as an adjunctive diagnosis of depression. This research was designed to evaluate whether fNIRS signals can differentiate different degrees of PSD and explore the pathogenesis behind PSD.

**Methods:**

We recruited 56 stroke patients treated at the Wenzhou TCM Hospital of Zhejiang Chinese Medical University and stratified them into three groups according to PSD severity: non-PSD (*n* = 18), mild-PSD (*n* = 19), and moderate-PSD (*n* = 19). fNIRS was employed to monitor frontotemporal cortical activity while administering a verbal fluency task across all participant groups. Differences in hemodynamic activity and functional connectivity across six frontotemporal cortex subregions were examined in three patient groups, and their correlations with 17-item Hamilton Depression Rating Scale (HAMD-17) scores were evaluated.

**Results:**

In terms of brain activation, the moderate-PSD group demonstrated significantly diminished activation in four particular brain regions in comparison to the non-PSD group (*p* < 0.05): the bilateral medial prefrontal cortex (mPFC), the ipsilateral dorsolateral prefrontal cortex (DLPFC), and the contralateral temporal lobe (TL), and the activation intensity within these regions was negatively associated with HAMD-17 scores (L-mPFC: r_s_ = −0.315, *p* = 0.018; R-mPFC: *r* = −0.377, *p* = 0.004; L-DLPFC: *r* = −0.323, *p* = 0.015; R-TL: *r* = −0.401, *p* = 0.002). Mild-PSD exhibited lower activation only in CH42 but higher in CH6 than moderate-PSD (*p* < 0.05). Regarding brain functional connectivity, the strength of connectivity between the DLPFC~mPFC on the ipsilesional side was positively correlated with the HAMD-17 scores (r_s_ = 0.405, *p* = 0.002), with significant disparities in the moderate-PSD versus the non-PSD groups. In contrast, the mild-PSD group displayed no notable connectivity differences between the two groups.

**Conclusion:**

This study presents distinct patterns of frontotemporal cortex activation and functional connectivity alterations associated with varying severity levels of PSD. In contrast with patients with stroke alone, PSD patients showed decreased activation levels and abnormally increased functional connectivity, and this change was more pronounced in moderate-PSD patients. These findings indicate that functional features of the frontotemporal cortex may serve as a neural indicator for identifying high-risk cases of PSD.

**Clinical trial registration:**

https://www.chictr.org.cn/showproj.html?proj=249555, ChiCTR2400093089.

## 1 Introduction

As indicated by epidemiological surveys, the occurrence, mortality, and relapse rates of stroke are rapidly increasing, driven by accelerated population aging and the widespread distribution of cerebrovascular risk factors, with the affected population notably becoming younger (1). Post-stroke depression (PSD) is a prevalent neuropsychiatric sequela following cerebrovascular accidents, and approximately 1/3 of people who have had a stroke will present with varying degrees of depressive symptoms (2). PSD affects not only patients’ physical, psychological, and social functioning but also slows down neural repair and reduces the motivation for rehabilitation (3). While the Hamilton Depression Scale (HAMD) is currently extensively utilized to assess depressive symptom severity, its assessment process often involves subjective factors and is susceptible to bias due to aphasia, cognitive impairment, and other factors, thus affecting the accuracy of diagnosis and treatment (4). Therefore, a deeper comprehension of the pathogenesis underlying different degrees of PSD is crucial for early detection and therapeutic interventions.

In today’s psychiatry, researchers commonly aim to identify depression-specific biomarkers with the help of advanced neuroimaging technologies to explore neurobiological mechanisms associated with depressive conditions (5, 6). Functional magnetic resonance imaging (fMRI), recognized as the prevailing benchmark in cerebral imaging, has provided important clues to unraveling the neural mechanisms of PSD. However, its inherent limitations, such as confined space, high noise level, high price, and sensitivity to subject motion, have limited its wide application in research (7). As a well-established, non-intrusive neuroimaging tool, functional near-infrared spectroscopy (fNIRS) has gained extensive application in investigating depression, schizophrenia, panic disorder, and other mental disorders (5). Compared with other functional brain imaging techniques (e.g., fMRI, MEG, EEG), it has both spatial and temporal resolution advantages (8), can effectively reduce motion artifacts, and has the characteristics of safety, low cost, no radiation, and portability (9). fNIRS can dynamically record changes in cortical hemodynamics by real-time monitoring of neural activity proximal to the cortical surface (10), thus providing brain activation levels and functional connectivity strengths that objectively reflect cortical excitability. Ho et al. (11) pointed out, based on a comprehensive analysis of 64 papers, that blood oxygen changes monitored by fNIRS can facilitate depression diagnosis, clinical symptom prediction, treatment response, and illness progression tracking.

In studies exploring the application of fNIRS for distinguishing depressed patients from healthy controls, most of the results show that patients’ depressive symptoms are closely associated with reduced hemoglobin oxyglobin (HbO) in the anterior cerebral cortex (12, 13), with the prefrontal lobe functioning as an essential mediator in affective regulation mechanisms (14, 15). However, no uniform conclusion has been reached regarding the intrinsic mechanism of prefrontal dysfunction triggered by depressive mood and the specific brain regions involved. Research has shown that PSD patients display diminished information processing capacity in the dorsolateral prefrontal cortex (DLPFC) while performing cognitive tasks (16). Nishizawa et al. (17) found, through the Stroop task, that depressed patients showed a specific brain region activation pattern compared to healthy controls: the left prefrontal cortex (PFC) was abnormally hyperactivated by negative lexical stimuli but hypoactivated by positive lexical stimuli. Akiyama et al. (18) found that depressed patients had reduced levels of bilateral frontotemporal lobe activation, with those with significant symptoms of low mood demonstrating a pronounced decrease in activation predominantly within the left frontotemporal area. At the same time, frontal lobe activation varies across depressive subgroups, which has been used by some researchers to distinguish confirmed depression from suspected depression (19).

The human brain is structured in an interconnected network-based organizational architecture, and therefore, analyzing brain network connectivity can help explore how the brain copes with focal injuries (20). Krick et al. (21) proposed that depression stems from the impairment of biogenic aminergic fiber bundles connecting the prefrontal lobe with other brain regions, that this disruption causes impaired functional network connectivity, and that depression severity is mainly associated with the right DLPFC and lesions in the inferior frontal gyrus. An fMRI-based study showed that PSD patients exhibited increased DLPFC connectivity with neural regions such as the bilateral lingual area, the contralateral superior frontal region, the precuneus, and the middle frontal area, revealing significant relationships between DLPFC-contralateral lingual area connectivity and the severity of depressive disorders (22). Currently, most investigations exploring functional connectivity in PSD are largely centered around resting-state assessments (23, 24), whereas few have investigated functional connectivity differences during task performance. However, Peng et al. (16) found that the task-related brain network has a significant advantage in distinguishing PSD and non-PSD patients by separately measuring and analyzing the DLPFC brain network characteristics under task execution and resting-state scenarios. The verbal fluency task (VFT) can effectively identify impairments in cognitive and executive functioning associated with the prefrontal regions of psychiatric patients (25, 26). Combined with the fNIRS technique, this paradigm has been extensively employed to identify a range of psychiatric disorders, such as bipolar disorder versus monophasic depression (27), initial-onset depression versus relapsing depression (28), and major depression versus generalized anxiety syndrome (29).

The value of the VFT-fNIRS approach in aiding the diagnosis of depression has been effectively validated (30). However, the variability in cerebral blood flow activation and functional connectivity and its correlation with the degree of depression in different PSD subgroups remains to be further elucidated. Based on this, this study sought to explore frontotemporal cortex activation and brain network characteristics in PSD patients across depression severity during VFT. We propose the following hypothesis: frontotemporal characteristics differ across stroke patients with varying levels of depression, and these differences can be visualized and characterized using fNIRS technology. The results are expected to reveal the pathogenesis of PSD patients and their association with depression severity from the frontotemporal perspective, thus providing a theoretical basis for diagnosing and delivering personalized therapy for PSD.

## 2 Materials and methods

### 2.1 Participants

This study recruited 60 stroke patients treated at Wenzhou TCM Hospital of Zhejiang Chinese Medical University from October 2024 to January 2025. Among them, 4 cases were excluded due to unqualified data quality. Inclusion criteria: (1) Unilateral cerebral hemisphere stroke diagnosed by MRI/CT (1–12 months duration), all lesion types/locations included; (2) Age 30–80 years, gender was not restricted; (3) Stable vital signs, conscious, cooperative for assessments; (4) Mini-Mental State Examination (MMSE) score of ≥ 20, able to follow instructions; (5) Right-handed. Exclusion criteria: (1) Patients in serious condition with unstable vital signs; (2) Impaired consciousness or severe communication disorders (including aphasia); (3) Post-cranioplasty patients; (4) Severe depression (HAMD-17 > 25); (5) Pre-stroke depression or recent antidepressant use (within 2 weeks). Before the start of the study, every participant was thoroughly informed about the experiment’s purposes, risks, process, and significance, and then provided written consent. Basic information about the participants was collected upon recruitment, comprising age, gender, stroke classification, illness duration, education level, and hemispheres involved, and patients were grouped according to their mood as assessed by the HAMD-17 scale: non-PSD: ≤ 7; mild-PSD: 8–17; moderate-PSD: 18–24. The study protocol was ethically approved by the Wenzhou TCM Hospital of Zhejiang Chinese Medical University (Approval No. WZY2024-KT-129-01) and was officially registered with the Chinese Clinical Trial Registry (Registration No. ChiCTR2400093089)^1^.

### 2.2 fNIRS introduction and data acquisition

**Figure 1:**
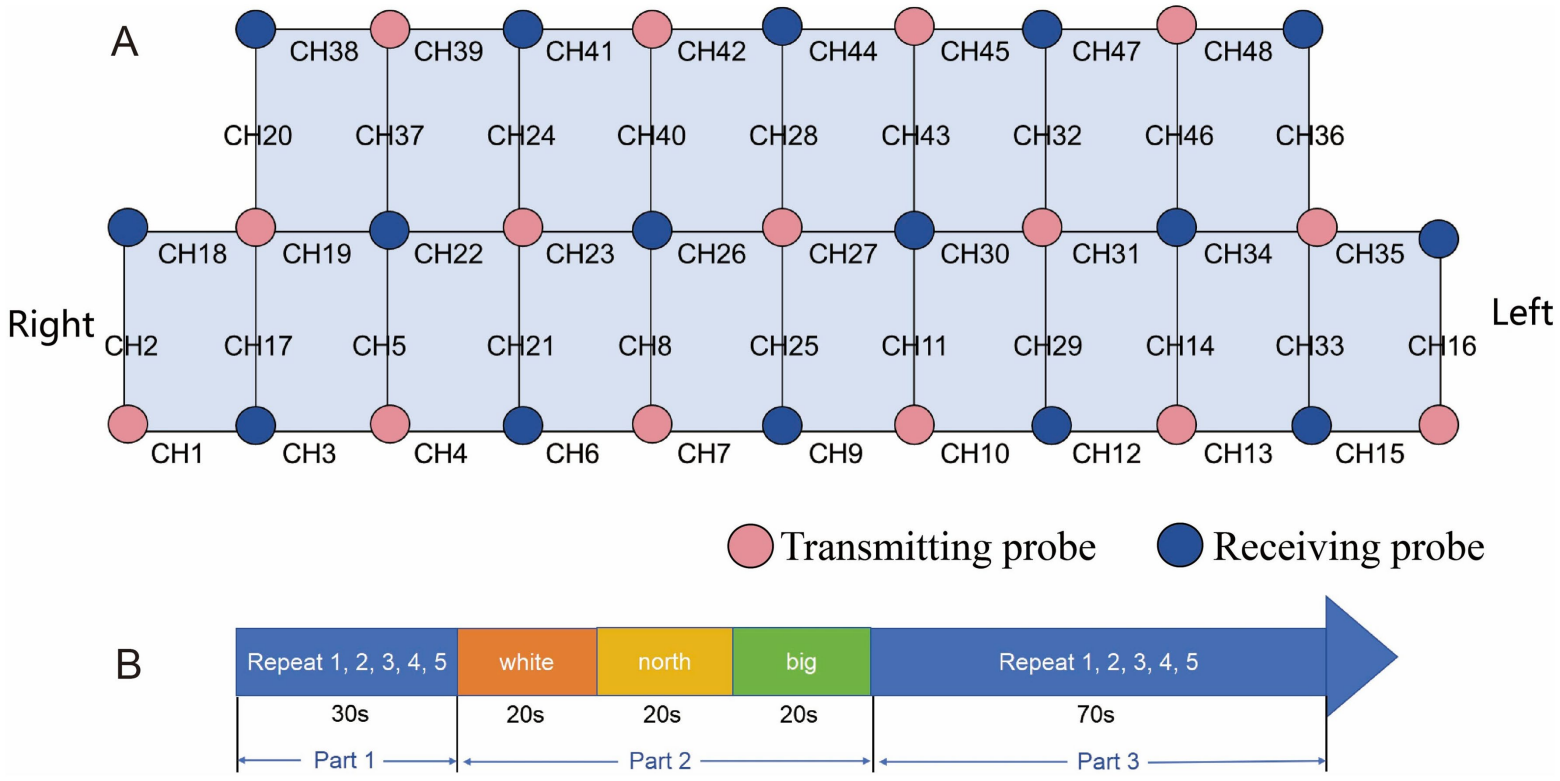
**(A)** The red circles denote 15 transmitting probes, while the blue circles indicate 16 receiving probes, with one channel formed between every two probes for 48 channels. **(B)** Example of VFT: a 30-s preparation phase (repeating numbers 1–5), a 60-s task phase (generating group words starting with “white,” “north,” and “big”), and a 70-s relaxation phase (repeating numbers 1–5 until completion).

This study employed a multichannel fNIRS system (Nirsmart-6000BS, Huichuang, Danyang, China) for real-time cortical activity monitoring. The system operated at three optical wavelengths (730 nm, 808 nm, and 850 nm) with a sampling frequency of 11 Hz, enabling continuous tracking and recording of fluctuations in oxygenated hemoglobin (Oxy-Hb), deoxygenated hemoglobin (Deoxy-Hb), and total hemoglobin (Total-Hb) concentrations (31). The monitoring system was equipped with 31 probes, including 15 transmitting probes and 16 receiving probes, with a probe spacing of 30 mm, which collaborated to form 48 channels for signal acquisition and analysis (Figure 1A). Participants were advised to ensure adequate sleep quality before testing and avoid consuming alcohol, coffee, or psychotropic medications. The experimental procedures were conducted in a quiet environment. When wearing the device, place the intermediate photoelectrode in the FPz position to ensure accurate positioning of the head cap. The entire VFT was divided into 3 parts (Figure 1B): Part 1 required participants to repeat the numbers 1–5; Part 2 required generating Chinese words from given characters (“white (白),” “north (北),” and “big (大)”) within 20-s intervals; and Part 3 required counting 1–5 repeatedly until the test ended. We confirmed that every subject comprehended the procedure before the assessment.

## 3 Data processing and analysis

### 3.1 Pre-processing

A total of 23 patients with right hemiplegia and 33 patients with left hemiplegia were included in this experiment. For patients with right-hemisphere stroke, hemodynamic signals were spatially normalized using Montreal Neurological Institute (MNI) coordinate-based mirror transformation (32, 33). This approach facilitates comparative analysis of interhemispheric symmetry and functional divergence, with the left hemisphere operationally defined as the ipsilesional side. Given that HbO typically exhibits a higher signal-to-noise ratio (26) and a strong correlation with local cerebral blood volume changes (34), all analyses in the present study were centered on HbO only.

**Figure 2:**
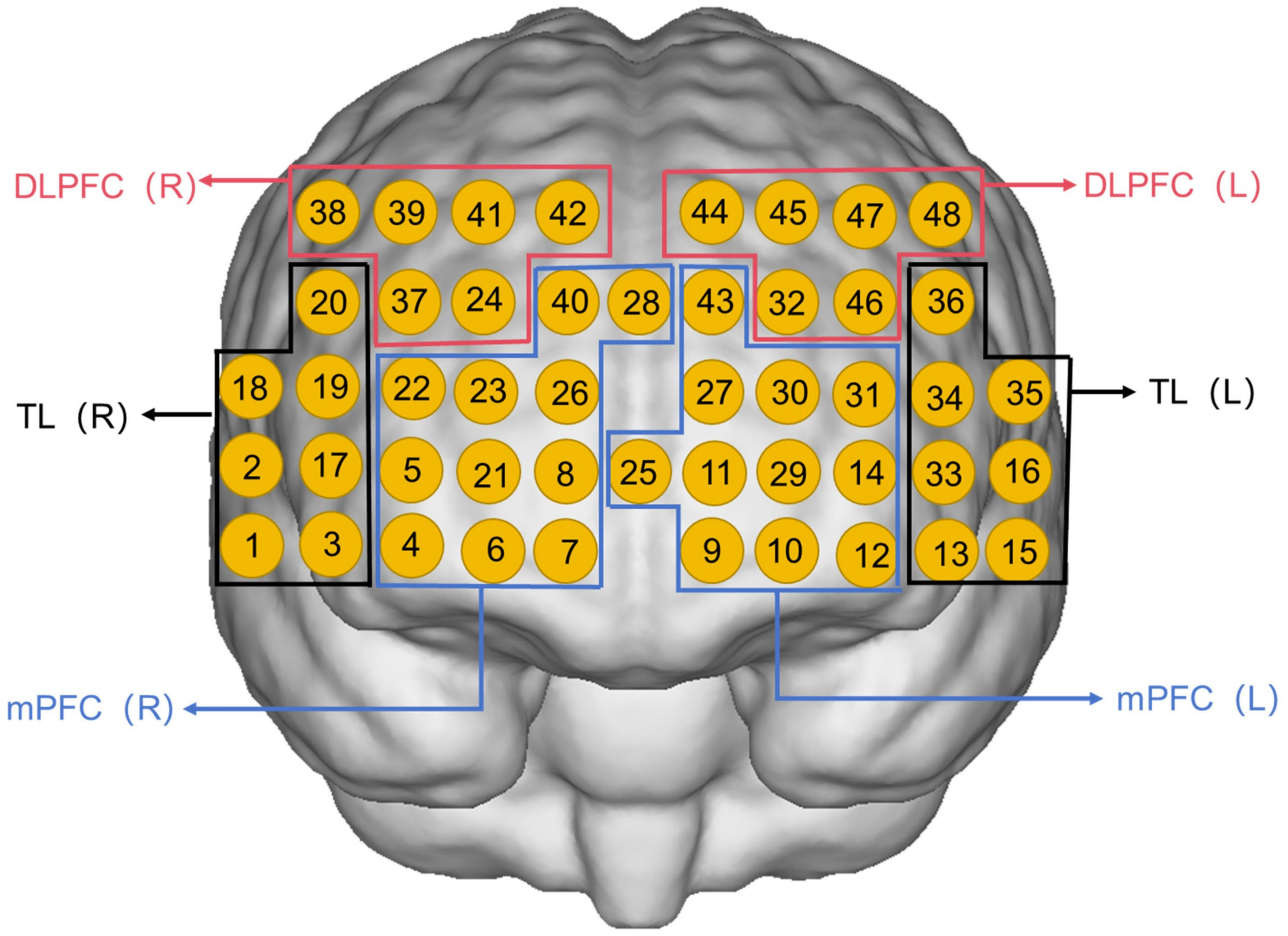
Channel arrangement of six regions of interest: left medial prefrontal cortex (L-mPFC) (Ch9, Ch10, Ch11, Ch12, Ch14, Ch25, Ch27, Ch29, Ch30, Ch31, Ch43); right medial prefrontal cortex (R-mPFC) (Ch4, Ch5, Ch6, Ch7, Ch8, Ch21, Ch22, Ch23, Ch26, Ch28, Ch40); left temporal lobe (L-TL) (Ch13, Ch15, Ch16, Ch33, Ch34, Ch35, Ch36); right temporal lobe (R-TL) (Ch1, Ch2, Ch3, Ch17, Ch18, Ch19, Ch20); left dorsolateral prefrontal cortex (L-DLPFC) (Ch32, Ch44, Ch45, Ch46, Ch47, Ch48); right dorsolateral prefrontal cortex (R-DLPFC) (Ch24, Ch37, Ch38, Ch39, Ch41, Ch42).

Preprocessing was performed in the preprocessing module of the NirSpark (Huichuang, Danyang, China) software in the following way: firstly, abnormal signal levels, excessive motion artifacts, and abnormal optical pole contact areas were removed by the naked eye, and then, the initial light intensity measurements were transformed into optical density (OD) values per channel, with motion artifacts were automatically corrected via the single-channel sliding-window method. The data were then processed with a 0.2 Hz low-pass filter to remove equipment noise and physiological artifacts (e.g., heartbeat, respiration). After that, the processed OD signals were converted into Oxy-Hb concentration variations following the adjusted Beer–Lambert law (35). We averaged the data from the three grouping modules on top of each other to obtain the average oxyhemoglobin value for each channel at 60 s in the task state. It has been suggested that averaging fNIRS metrics within brain region channels is more reliable compared to averaging at the single channel level (36), so in this study, the 48 channels were divided into six regions of interest (ROIs) following the Brodmann partitioning (37) (Figure 2): left and right DLPFC; left and right medial prefrontal cortex (mPFC); left and right temporal lobe (TL). Pairwise functional connectivity is constructed by calculating the Pearson correlation coefficient r between the time series of the ROI, performing a Fisher-z transformation to convert it into a z-score, and taking the average to form a 6*6 connectivity matrix (38).

### 3.2 Statistical analysis

Demographic information and pre-processed data were analyzed utilizing IBM SPSS Statistics 25.0. Categorical data were analyzed through the chi-square test and presented as frequencies (%). Quantitative data were first evaluated for normality (Shapiro–Wilk) and homogeneity of variance (Levene’s test). One-way ANOVA with LSD correction was applied to normally distributed data, while the Kruskal-Wallis H-test with Bonferroni correction was employed for non-normally distributed data. False discovery rate (FDR) (39) with a maximum threshold of 0.05 was applied to adjust for multiple comparisons across channels. Statistical significance was characterized as *p* < 0.05. Correlations between measures of brain function, HAMD scores, and stroke localization were determined using Pearson (normal distribution) or Spearman (non-normal distribution) tests. Subgroup and interaction analyses were applied to examine the potential modifying effects of variables such as age, gender, and stroke type.

## 4 Results

### 4.1 Demographic analysis

All participants’ demographic and clinical profiles are summarized in Table 1, encompassing 18 in the non-PSD group and 19 in both the mild-PSD and moderate-PSD groups. No significant statistical variations were observed among the three groups regarding age, sex, educational background, stroke type, disease duration, affected hemisphere, and the number of group words (*p* > 0.05). HAMD-17 scores differed among the three groups (*p* < 0.001).

**Table 1:** Demographic and clinical information of subjects.

Demographic characteristics	Non-PSD (*n* = 18)	Mild-PSD (*n* = 19)	Moderate-PSD (*n* = 19)	X^2^/F/H	*p*-value
Age (years)	53.50 (20.25)	65 (24)	70 (18)	4.649	0.098^c^
Gender [*N* (%)]				1.635	0.442^a^
Male	12 (66.7%)	9 (47.4%)	12 (63.2%)		
Female	6 (33.3%)	10 (52.6%)	7 (36.8%)		
Education(years)	5.5 (7.25)	9 (6)	6 (8)	5.293	0.071^c^
Stoke type [N (%)]				0.065	0.968^a^
Ischemic	12 (66.7%)	12 (63.2%)	12 (63.2%)		
Hemorrhage	6 (33.3%)	7 (36.8%)	7 (36.8%)		
Onset time (days)	60 (92)	30 (40)	30 (100)	1.787	0.409^c^
Stroke location				0.500	0.779^a^
Cortical	6 (33.3%)	7 (36.8%)	5 (26.3%)		
Subcortical	12 (66.7%)	12 (63.2%)	14 (73.7%)		
Hemisphere of lesion [*N* (%)]				4.401	0.111^a^
Left	11 (61.1%)	6 (31.6%)	6 (31.6%)		
Right	7 (38.9%)	13 (68.4%)	13 (68.4%)		
Number of words	5.56 ± 2.57	5.68 ± 2.70	4.68 ± 3.48	0.643	0.530^b^
MMSE	25 (5)	28 (5)	26 (5)	0.580	0.748^c^
HAMD-17	4 (4)	9 (4)	18 (2)	49.741	<0.001^c^

The values are expressed as mean ± SD, median (IQR), or N (%).

MMSE: Mini-Mental State Examination; HAMD-17: 17-item Hamilton Depression Rating Scale.

HAMD-17 scores corrected for Bonferroni multiple comparisons: Non-PSD vs. Mild-PSD (*p* = 0.002), Non-PSD vs. Moderate-PSD (*p* < 0.001), Mild-PSD vs. Moderate-PSD (*p* = 0.001).

^a^Chi-square test; ^b^One-way analysis of variance test; ^c^Kruskal-Wallis H-test.

### 4.2 Brain activation characterization

**Figure 3:**
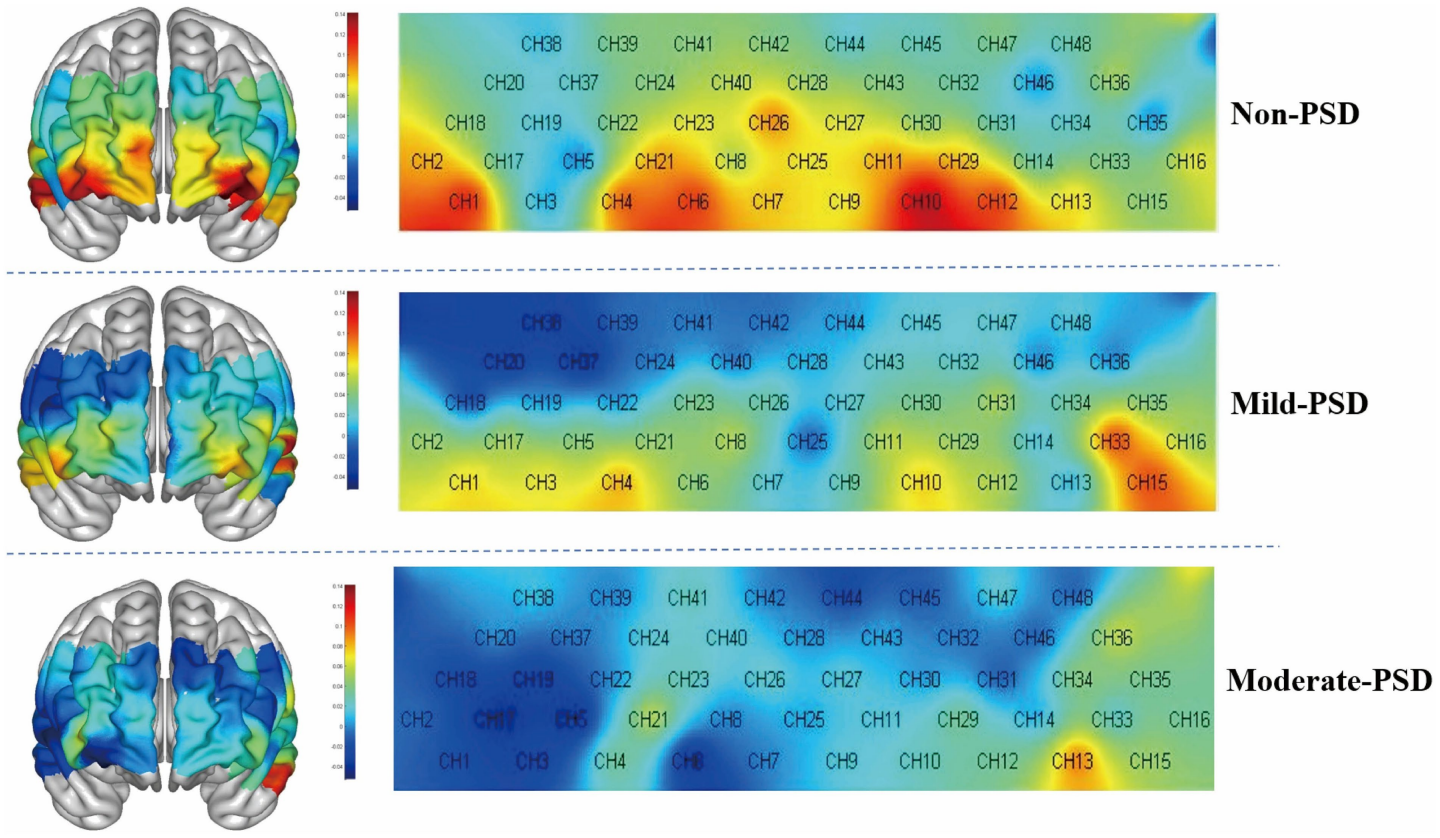
3D and 2D plots of frontotemporal activation during the VFT for the three groups, with redder colors representing greater means.

**Figure 4:**
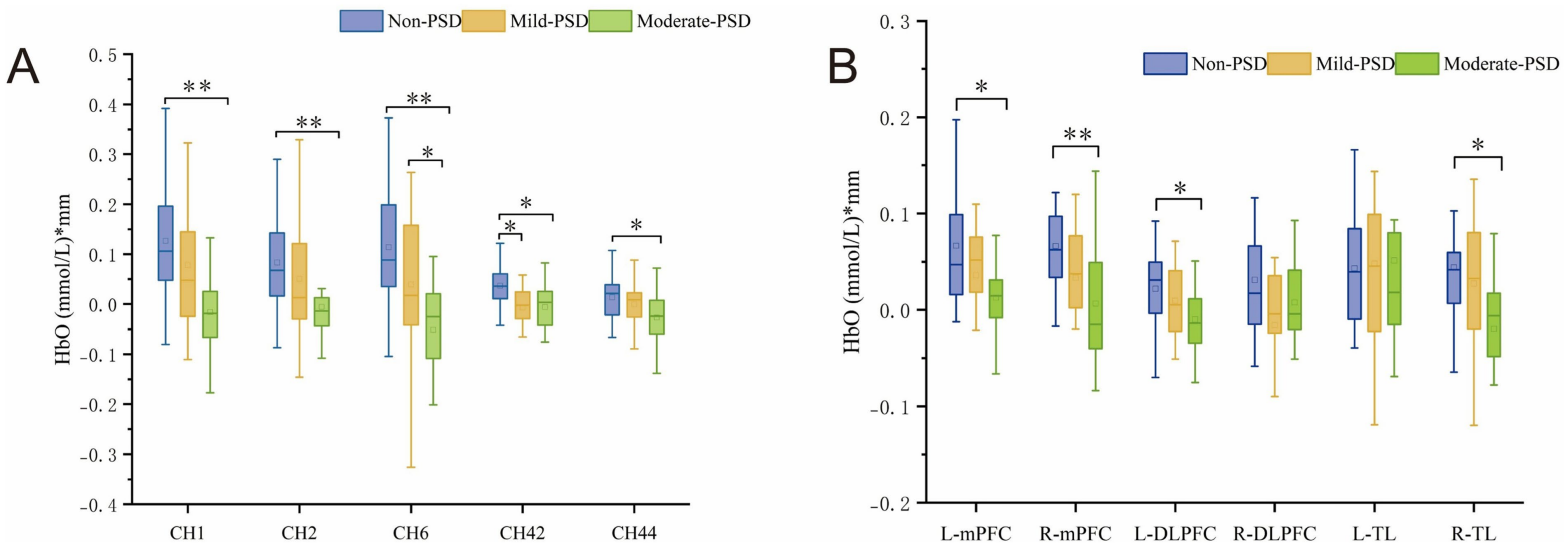
**(A)** Channels showing significant differences at the single-channel level in Non-PSD, Mild-PSD, and Moderate-PSD groups, *p < 0.05, **p < 0.01. **(B)** Degree of activation in Non-PSD, Mild-PSD, and Moderate-PSD groups at the level of six ROIs, *p < 0.05, **p < 0.01.

**Figure 5:**
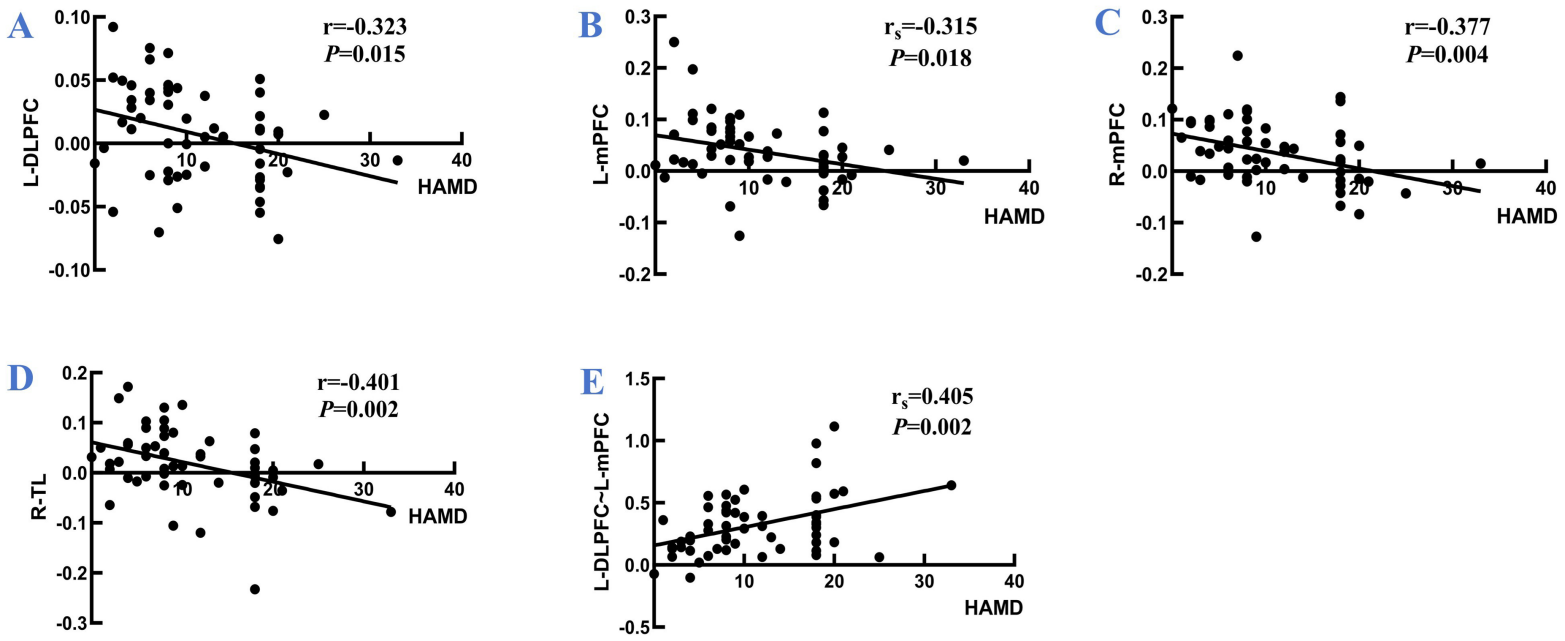
Panel **(A–D)** are the correlations between the activation levels of each brain region of L-DLPFC, L-mPFC, R-mPFC, and R-TL and the HAMD scale scores for all participants, respectively; **(E)** illustrates the correlation between the *z*-value of the strength of the L-DLPFC~L-mPFC interconnections and the HAMD scale scores for all participants. r: Pearson’s correlation coefficient; r_s_: Spearman correlation coefficient.

During the VFT, significant variations in frontotemporal cortex activation were noted across the three subject groups (Figure 3): the overall frontotemporal cortex activation level in the moderate-PSD group was reduced compared to the other two groups, and the mild-PSD group showed lower activation than the non-PSD group. Analyzing from the single-channel dimension, the HbO hemodynamic responses of the moderate-PSD group were notably reduced compared to those of the non-PSD group (corrected *p* < 0.05) in six channels, namely, CH1, CH2, CH6, CH42, and CH44 (Figure 4A), which mainly corresponded to the R-TL (CH1, CH2), R-mPFC (CH6), R-DLPFC (CH42), and L-DLPFC (CH44) four brain regions. In addition, the mean HbO values in CH6 were also markedly lower in the moderate-PSD group than in the mild-PSD group (corrected *p* < 0.05). In contrast, the mild-PSD group showed lower activation than the non-PSD group only in the CH42, and the data distribution between the two groups was more evenly distributed in the remaining channels. Analyzed at the ROIs level, except for L-TL and R-DLPFC brain regions, the mean HbO values of the remaining regions of interest were significantly different when contrasting the moderate-PSD group with the non-PSD group (corrected *p* < 0.05) (Figure 4B), and the activity levels of these brain regions revealed a significant inverse relationship with the HAMD-17 scores (L-DLPFC: *r* = −0.323, *p* = 0.015; L-mPFC: r_s_ = −0.315, *p* = 0.018; R-mPFC: *r* = −0.377, *p* = 0.004; R-TL: *r* = −0.401, *p* = 0.002) (Figures 5A–D).

### 4.3 Brain network characterization

**Figure 6:**
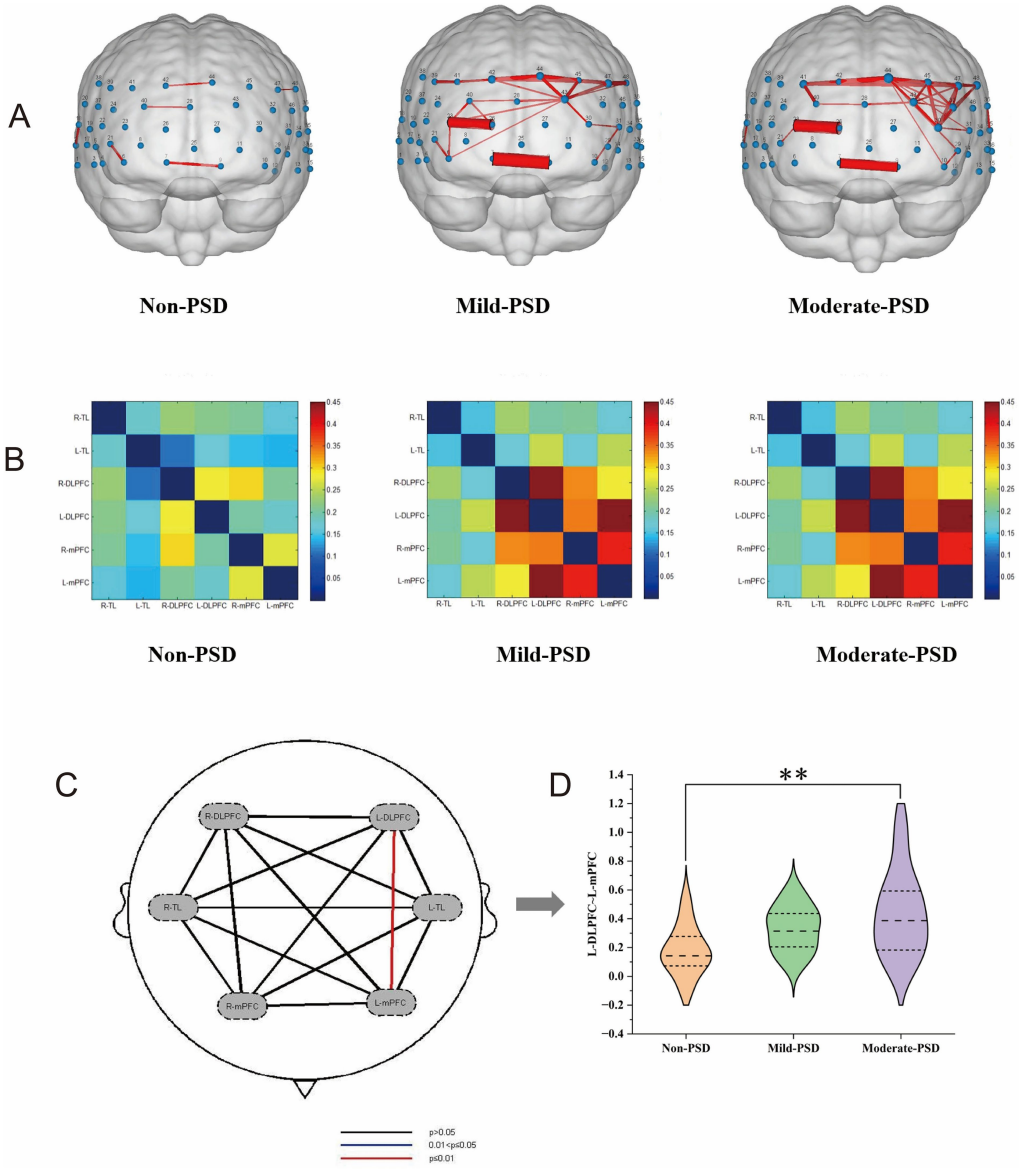
**(A)** Line width represents inter-channel correlation strength (*z*-value), with thicker lines indicating stronger connectivity. **(B)** Matrix axes correspond to ROI groups, with cell coloration representing the strength of the connection between the two. **(C)** Shows the findings from the statistical comparison of the three groups across any two regions of interest. **(D)** Represents the functional connectivity values *z* between L ~ DLPFC-L ~ mPFC for the three groups, **p < 0.01.

NirSpark software was employed to characterize the variations in functional connectivity across the brain networks of the three groups of cases during VFT, and the channel similarity threshold was set to 0.5 (i.e., functional connectivity values greater than 0.5 were considered as valid connectivity, and those less than 0.5 were excluded). The results showed that the average local frontotemporal cortex channel connectivity strength was stronger in the mild and moderate PSD groups, mainly concentrated in CH7, CH9, CH23, CH26, CH40, CH41, CH42, CH43, CH44, CH45, CH47, and CH48, which differed significantly from the non-PSD group, whereas the functional connectivity patterns of the mildly depressed and moderately depressed groups showed high similarity (Figure 6A). Corresponding to the ROIs, the connection strengths of L-DLPFC~L-mPFC and L-DLPFC~R-DLPFC were mainly manifested in the depressed group, demonstrating markedly greater strength compared to the non-PSD group (Figure 6B); one-way ANOVA further revealed that the connection strengths between L-DLPFC~L-mPFC differed significantly across the three groups (corrected *p* < 0.01), in which, the moderate-PSD group exhibited notably stronger than the non-PSD group (corrected *p* < 0.01) (Figures 6C,D). Spearman correlation analysis showed that the connectivity strength between the L-DLPFC and L-mPFC was markedly and positively linked to the HAMD scores (r_s_ = 0.405, *p* = 0.002) (Figure 5E). While no notable differences were observed between the mild-PSD and non-PSD in this analysis, in Figure 6B, it is shown that the connectivity was highly similar between the mild-PSD and moderate-PSD groups, with both trending nearly the same.

### 4.4 Correlation with stroke location

To investigate whether fNIRS signals are intrinsically associated with stroke topography, we analyzed their correlations with cortical and subcortical lesions. Statistical analyses revealed no significant correlations between stroke location and either brain activation or functional connectivity (all *p* > 0.05) (Table 2). Similarly, lesion localization showed no correlation with HAMD-17 scores (all *p* > 0.05) (Table 2). These results indicate that fNIRS signals and depression severity are not affected by the depth of the lesion location.

**Table 2:** Regional signal and HAMD score correlations with stroke location.

Stroke location	Analysis category		Correlation coefficient(r_s_)	*p*-value
Cortical/Subcortical	Functional connectivity	R-TL ~ L-TL	0.031	0.822
		R-TL ~ R-DLPFC	0.028	0.835
		R-TL ~ L-DLPFC	0.071	0.603
		R-TL ~ R-mPFC	0.097	0.477
		R-TL ~ L-mPFC	0.076	0.579
		L-TL ~ R-DLPFC	0.097	0.477
		L-TL ~ L-DLPFC	−0.083	0.544
		L-TL ~ R-mPFC	0.147	0.281
		L-TL ~ L-mPFC	−0.114	0.405
		R-DLPFC~L-DLPFC	0.128	0.348
		R-DLPFC~R-mPFC	0.118	0.385
		R-DLPFC~L-mPFC	0.196	0.147
		L-DLPFC~R-mPFC	0.064	0.640
		L-DLPFC~L-mPFC	0.064	0.640
		R-mPFC~L-mPFC	0.028	0.835
	Brain activation	R-TL	−0.246	0.068
		L-TL	−0.263	0.051
		R-DLPFC	−0.021	0.876
		L-DLPFC	0.019	0.890
		R-mPFC	−0.054	0.690
		L-mPFC	−0.111	0.415
	HAMD-17		0.112	0.411

### 4.5 Subgroup analysis

**Figure 7:**
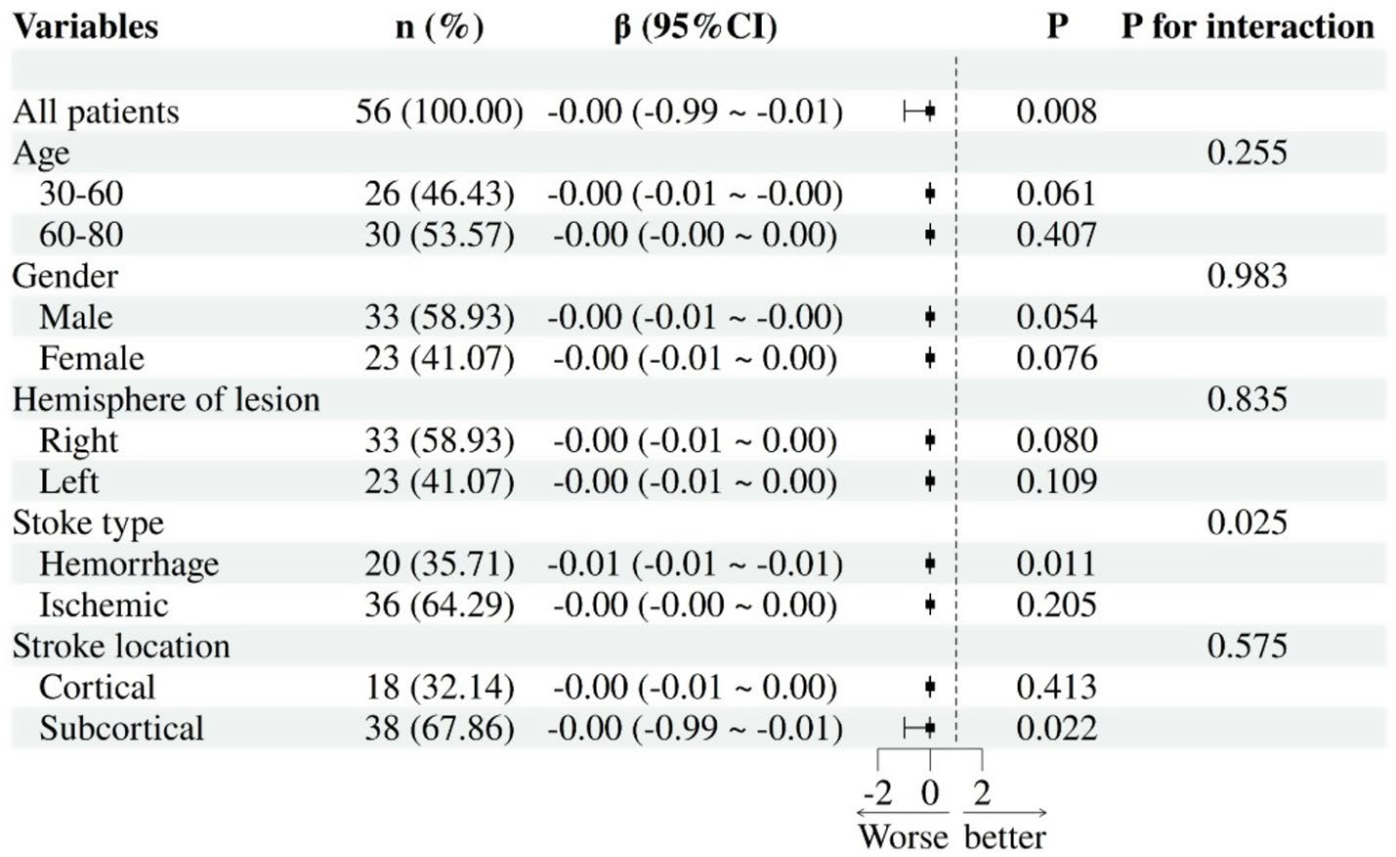
Subgroup analysis: association between HAMD-17 scores and L-mPFC activation in PSD.

This study employed multiple linear regression analysis, with HAMD score as the independent variable and statistically significant brain area indicators (including L-DLPFC, L-mPFC, R-mPFC, R-TL activation level, and L-DLPFC-L-mPFC functional connectivity) as dependent variables. All models were adjusted for covariates like MMSE and years of education. Subgroup analyses were conducted by age (30–60/60–80), gender, stroke type (hemorrhagic/ischemic), and stroke location (cortical/subcortical) to explore subgroup differences in depression-frontotemporal activity associations. The results showed that the negative correlation between HAMD score and L-mPFC activation was more significant in patients with hemorrhagic stroke (*β* = −0.01, 95% CI: −0.01 to −0.01, *p* = 0.011) (Figure 7). Further analysis showed that age (*p* = 0.255), gender (*p* = 0.983), affected hemisphere (*p* = 0.835), or stroke location (*p* = 0.575) did not significantly modulate the association between HAMD score and L-mPFC (interaction > 0.05). Additionally, the correlation between HAMD score and L-DLPFC, R-mPFC, R-TL, L-DLPFC~L-mPFC was not adjusted by demographic and clinical characteristics (*p* > 0.05 for each interaction).

## 5 Discussion

### 5.1 Brain activation

This study employed fNIRS to measure HbO levels in the frontotemporal lobes and found that while the three participant groups showed no significant difference in word generation counts during the VFT, compared to the non-PSD group, the moderate-PSD group exhibited widespread reduced hemodynamic responses across multiple frontotemporal regions (including the ipsilesional DLPFC, bilateral mPFC, and contralesional TL). In contrast, the mild-PSD group showed reduced regional activation only in the contralesional DLPFC (CH42). Furthermore, the mild-PSD group exhibited higher activity in the contralesional mPFC (CH6) than the moderate-PSD group. This graded impairment pattern suggests that the neuropathology of PSD may follow a “compensatory-decompensatory” two-stage model: during the mild depression stage, insufficient compensatory activation occurs in the unaffected DLPFC (CH42), while the unaffected mPFC (CH6) can still partially maintain function; during the moderate depression stage, metabolic dysregulation emerges in multiple frontotemporal regions. This finding is partially consistent with Li′s study (40), which reported prefrontal triangularis abnormalities exclusively in mild PSD.

Koyanagi et al. (41) discovered that PSD participants exhibited markedly decreased integral values of Oxy-Hb in the frontal lobe, which negatively correlated with the total HAMD-17 score, revealing a close association between depression severity and reduced prefrontal HbO. Sun et al. (42) further noted that the lower oxygen activation in the DLPFC among depressed individuals results from an inability to obtain sufficient blood supply to meet metabolic demands, especially in refractory depressed patients, who showed significant hypoactivation in both the mPFC and DLPFC brain regions. In addition, some studies have found that depressed patients have lower frontotemporal lobe cerebral hemodynamic responses to the VFT, which may be intimately linked to mitochondrial dysfunction, emphasizing the critical significance of brain metabolic regulation in depression (43). The results of this series of studies indicate that PSD patients not only exhibit physical dysfunction and depressed mood but also involve deeper issues of brain energy metabolism and blood oxygen supply. Hypoactivation of the frontotemporal lobe may be one of the core neurobiological features of PSD patients, and this alteration is closely related to the severity of depressive symptoms and neurometabolic dysfunction.

### 5.2 Brain network

Most brain imaging research on depression has concentrated on resting-state functional connectivity, interpreting the language system as a static architecture (44). However, the brain operates as a complex and dynamically interacting system, with the language network exhibiting inherent dynamism. Monitoring the dynamic reorganization of the language system may demonstrate higher predictive validity than traditional static measures, revealing more core characteristics of cognitive activity (45, 46). Consequently, this study employed the VFT to calculate the functional connectivity within the ROIs. Results demonstrated significantly enhanced DLPFC~mPFC functional connectivity in the ipsilesional hemisphere of moderately depressed patients, with connectivity strength positively correlating with depression severity. Mechanistically, this hyperconnectivity may reflect compensatory neuroplasticity, wherein stroke-induced neural damage triggers aberrant functional excitation within networks, resulting in disinhibition (47). Padmanabhan et al. (48) proposed and verified that the left DLPFC is the center of the depressive circuitry, with its functional connectivity to the lesion site being strongly linked with depression (22), regardless of the specific location or size of the PSD lesion. Consistent with the findings of Padmanabhan et al., the present research yielded no substantial evidence for associations between stroke lesion location and HAMD scores. This further suggests that the mechanism of PSD may be more dependent on the dynamic functional reorganization of the network than on local structural damage.

Functionally, the mPFC and DLPFC are pivotal for emotion regulation. Dysfunction in these regions, caused by neurobiological abnormalities, neurotransmitter imbalances, or dysregulated neural connectivity, leads to impaired mood regulation and disrupted interactions between cognitive functions (e.g., attention, memory, executive function) and mood (49, 50). Neurotoxic mechanisms (e.g., inflammation and oxidative stress) are triggered during the onset of depression and anxiety, causing reductions in hippocampal and mPFC volumes (50). If excitatory brain stimulation is applied to the DLPFC, the neural activity in this brain region can be modulated, which effectively reduces depressive symptoms and improves executive function (51).

Depression has been reported to correlate with heightened connectivity across the default mode network (DMN, encompassing the mPFC and posterior cingulate cortex) and the left DLPFC region (52). Enhanced functional coupling between the DMN and the frontoparietal network (FPN, covering the DLPFC, inferior parietal sulcus, and posterior parietal cortex) characterizes patients engaged in reflective thinking and showing a lack of attention to the outside environment. Furthermore, the DMN is downregulated in attention-neutral tasks and upregulated during thinking and memory-related activities (53). In addition, some researchers (54, 55) have proposed that the increased resting-state functional connectivity of the mPFC observed in depression is attributable to the relative hypermetabolism of this brain area and that the metabolic activity of this region decreases accordingly after treatment. However, some researchers have reached different conclusions. For example, Wu X. et al. (20) applied fMRI to explore the differences in PSD brain networks during the resting state and discovered that the functional connectivity within the robust angular gyrus, posterior cingulate cortex, and hippocampus was markedly enhanced in individuals with PSD. On the other hand, Zhang et al. (56) concluded that both functional and structural brain connectivity decreased in PSD patients, with disrupted coupling between the two. The strength of channel connectivity and the differences involving specific brain regions observed in these studies usually result from a combination of intricate factors, among which the choice of task paradigm is an extremely critical factor. In addition, individual differences in experimental participants, sample size, limitations in research methodology, and potential confounding variables may also lead to completely different results from different studies. Nevertheless, collectively these findings provide evidence that the dysregulation of functional brain networks in patients with PSD reflects complex and multidimensional alterations in functional connectivity, which may vary across individuals or disease stages.

Following the stroke, perilesional cortical connectivity is frequently disrupted (57, 58). The PFC region that forms a network linked with cortical and subcortical structures (51) is pivotal in emotion regulation and cognitive management (59). Given this pivotal role, the prevalent depressive symptoms in PSD likely reflect PFC-mediated circuit dysfunction. Therefore, we hypothesize that the abnormal enhancement of connectivity in the ipsilesional hemisphere in the present study may represent a key feature of PSD pathophysiology and that the brain compensates for the dysfunctions triggered by stroke and depression to a certain extent by strengthening the frontal cortical connectivity with low connectivity strength to achieve effective regulation of emotion and cognition. However, the neurobiological mechanisms and functional implications of this compensatory process require further elucidation through multimodal neuroimaging combined with behavioral phenotyping.

Whether examining brain activation or neural network connectivity, fewer significant differences were observed between the mild-PSD group and the moderate-PSD and non-PSD groups, a result that might be explained by the restricted capacity of the HAMD scale to differentiate the severity of depression (25), resulting in less precise grouping; or by the reality that there is a certain level of insensitivity in detecting mild PSD with the fNIRS technique. Despite this, the fNIRS signals demonstrated a notable association with depression severity in PSD patients. This technique holds promise for the early assessment of depression intensity, particularly in examining individuals with moderate to severe depressive symptoms, and may provide auxiliary support for clinical diagnosis and condition assessment.

### 5.3 Subgroup

This study also examined how gender, age, and other factors influence the activation and interconnection of the key brain regions involved in PSD. In the L-mPFC region, the negative correlation between HAMD scores and mPFC activation was significantly stronger in patients with hemorrhagic stroke compared to those with ischemic stroke. This aligns with reports of a higher PSD risk for hemorrhagic stroke, which is characterized by more frequent anxiety, loss of interest, insomnia, and fatigue (60, 61). Bleeding triggers a biological response that causes an imbalance of multiple hormones, pro-inflammatory cytokines, neurotransmitters, and neurotrophic factors (2). Clinical data show that up to 70% of patients with frontal lobe hemorrhage will develop depression, and changes in L-mPFC activity are closely linked to depressive-like behaviors (62, 63). It should be noted that the confidence interval of this result is very narrow (interval width < 0.01), and its clinical significance requires further verification through larger sample studies. Additionally, age, gender, and lesion hemisphere or location did not significantly affect the HAMD-mPFC relationship, suggesting this connection may be consistent across different populations. These findings support the “prefrontal suppression hypothesis of hemorrhagic PSD” and pave the way for exploring mPFC-targeted neuromodulation (such as transcranial magnetic stimulation) and stratified treatment strategies that incorporate biomarker detection in the future.

## 6 Summary

Our study revealed that frontotemporal cortex activation was generally reduced in the moderate-PSD group, whereas the mild-PSD group exhibited only a slight reduction. Concurrently, connectivity between the ipsilesional DLPFC and mPFC significantly strengthened with worsening depression severity. Notably, these depression-related signaling changes were uninfluenced by factors such as age, gender, or stroke location. Collectively, these results establish the potential of using the fNIRS technique to investigate the cortical mechanisms underlying PSD. We further propose that frontotemporal cortical signaling alterations may be a potential neural mechanism of PSD, and future studies should pay more attention to the functional changes of frontotemporal regions in PSD and explore ways to alleviate depressive symptoms by improving brain metabolism, enhancing blood flow supply, or restoring neuroplasticity.

## 7 Limitations

Our findings revealed distinct changes in frontotemporal activation and neural networks in individuals with PSD of different severities, but some limitations remain. First, we enrolled a small number of participants, which limited the generalizability of the results and may be one of the reasons why the mild-PSD group differed less from the remaining two groups. Second, residual confounding may persist due to unmeasured variables such as stroke lesion volume and comorbid conditions. Third, the present research employed a cross-sectional approach, lacking ongoing monitoring and assessment of the treatment process, which did not effectively tap into the potential predictors of disease prognosis. Future research should adopt larger sample sizes and longitudinal designs while systematically controlling for potential confounding factors to validate these findings. Fourth, fNIRS technology exhibits limited penetration depth (typically < 3 cm from the scalp), restricting its capacity to monitor subcortical structure, and the skull thickness influences the sensitivity of the signals (64). With technological advances, it is hoped that by integrating multimodal neuroimaging techniques in the future, we will be able to further accurately map the dynamics of signaling changes in the frontotemporal cortex during the occurrence and progression of PSD, identify specific markers of the disease’s neural mechanisms, and establish a novel approach for early identification and monitoring of PSD. This will deliver fresh targets and approaches for early diagnosis, disease surveillance, and personalized interventions for PSD.

## Data Availability

The raw data supporting the conclusions of this article will be made available by the authors, without undue reservation.

## Footnotes

^1^ https://www.chictr.org.cn/showproj.html?proj=249555
